# Social media use duration and epigenetic aging among U.S. adults in the MIDUS refresher study

**DOI:** 10.1371/journal.pdig.0001570

**Published:** 2026-08-06

**Authors:** Mariana Rodrigues, Jemar R. Bather, Alisha A. Crump, Emiko O. Kranz, Kimberly A. Kaphingst, David M. Almeida, Adolfo G. Cuevas

**Affiliations:** 1 Department of Social and Behavioral Sciences, New York University School of Global Public Health, New York, New York, United States of America; 2 Department of Biostatistics, New York University School of Global Public Health, New York, New York, United States of America; 3 Huntsman Cancer Institute, University of Utah, Salt Lake, Utah, United States of America; 4 Department of Communication, University of Utah, Salt Lake, Utah, United States of America; 5 Department of Human Development and Family Studies, Pennsylvania State University, University Park, Pennsylvania, United States of America; 6 Center for Healthy Aging, Pennsylvania State University, University Park, Pennsylvania, United States of America; Harvard Medical School, UNITED STATES OF AMERICA

## Abstract

Social media platforms are central to daily social interaction, with emerging evidence linking higher engagement to adverse outcomes, including increased stress, sleep disturbance, and depressive symptoms. However, whether these digital exposures translate into measurable acceleration of biological aging remains unexplored, particularly among midlife and older adults. We analyzed cross-sectional data from 323 participants (Mean age = 47.5 years, SD = 11.8) in the Midlife in the United States Refresher Study who completed daily diary assessments of social media use duration and provided blood samples for epigenetic aging measurement. Epigenetic aging was quantified using DunedinPACE and GrimAge2 from genome-wide DNA methylation analysis. In a linear regression model controlling for differences in inter-assessment intervals, sociodemographic characteristics, health behaviors, and self-reported mental health, each additional hour of daily social media use was significantly associated with a 0.19-SD higher DunedinPACE score (95% CI: 0.08 to 0.29, *p* < 0.001). However, further adjustment for body mass index attenuated this association (0.09 SD increase, 95% CI: -0.01, 0.19, *p* = 0.088), suggesting that BMI may reflect the downstream consequences of broader behavioral, metabolic, psychosocial, environmental, and health-related processes relevant to the observed relationship. No associations were observed with GrimAge2 across any model. These findings provide preliminary evidence that social media use duration is associated with the pace of biological aging as measured by DunedinPACE. Future research should employ longitudinal designs with direct measurement of digital engagement, behavioral and psychosocial processes, and physiological mechanisms to clarify how digital environments may relate to biological aging over time.

## Author summary

Social media is part of everyday life for many people, yet researchers still know little about how it may relate to long-term physical health and aging. In this study, we examined whether the amount of time adults spent using social media was associated with epigenetic aging, a molecular indicator of biological aging based on DNA methylation patterns. DNA methylation refers to chemical marks on DNA that help regulate gene activity and can change with age, stress, behavior, and other exposures. We analyzed data from adults participating in the Midlife in the United States (MIDUS) Refresher Study who completed repeated daily diary assessments of social media use and provided blood samples for DNA methylation analysis. We examined two next-generation epigenetic aging measures: DunedinPACE, which captures the pace of biological aging, and GrimAge2, which reflects mortality- and disease-related aging risk. Greater social media use duration was associated with a faster pace of biological aging as measured by DunedinPACE, although this association was attenuated after accounting for body mass index. No association was observed for GrimAge2. Together, these findings contribute to emerging research examining how digital environments may relate to biological aging and long-term health processes.

## Introduction

Social media platforms are central features of contemporary social life, shaping how individuals communicate, seek support, and engage with broader social networks [1,2]. These environments can foster connection, belonging, and access to social support; all of which may support psychological well-being [3–5]. At the same time, social media platforms may also expose users to chronic psychosocial stressors, including social comparison, discrimination, harassment, and negative social evaluation [6–8]. These digitally mediated experiences may shape emotional well-being and stress-related processes, particularly when interactions are frequent, socially evaluative, or difficult to disengage from [9–11].

Indeed, emerging evidence links higher social media engagement with elevated perceived stress, sleep disturbance, and depressive symptomatology, all of which are intermediate risk factors for accelerated aging—that is, biological changes that outpace chronological age, reflecting faster physiological decline and increased vulnerability to age-related diseases and mortality [12–16]. However, it remains unclear whether social media use is associated with measurable indicators of biological aging, particularly among midlife and older adults. DNA methylation-based epigenetic aging biomarkers offer one approach for examining this question by capturing molecular signatures associated with aging-related physiological change [17,18]. In particular, DunedinPACE and GrimAge2 capture distinct dimensions of biological aging, with DunedinPACE reflecting the pace of biological aging and GrimAge2 capturing morbidity- and mortality-related methylation patterns [17,18].

Both behavioral and psychosocial pathways may contribute to associations between social media use and biological aging [13,19,20]. Health behaviors such as substance use and sedentary activity, each associated with social media use, represent established correlates of stress and aging [13,19,20]. Sedentary behavior, in particular, has been associated with metabolic dysregulation and chronic inflammation, both linked to epigenetic age acceleration [21–23]. In parallel, psychosocial processes linked to digital engagement, including hypervigilance, perceived social threat, and upward social comparison, may operate through overlapping stress-related neuroendocrine and inflammatory mechanisms [24–26]. Although these pathways are theoretically plausible, they remain understudied in relation to epigenetic aging, and most existing research on social media and health has focused on psychological or behavioral outcomes rather than molecular biomarkers [27,28].

The present study investigates associations between social media use duration and epigenetic aging as measured by DunedinPACE and GrimAge2 in a national sample of midlife and older adults from the Midlife in the United States (MIDUS) Refresher study. This novel assessment focusing on duration of social media use provides a first step toward understanding whether digital social environments are associated with biologically embedded aging processes. In doing so, we conceptualize social media use not merely as individual behavior but as a consequential feature of the contemporary social environment.

## Methods

### Ethics statement

All MIDUS protocols were approved by the University of Wisconsin Institutional Review Board. This secondary analysis of publicly available, de-identified data did not require additional IRB approval from New York University. Data were accessed for research purposes on 25/09/2025. The IRB waived the informed consent requirement.

### Study design, setting, and sample

This cross-sectional study used data from the Midlife in the United States (MIDUS) Refresher Study, a national study of U.S. adults aged 25–75 years [29]. From 2011 to 2014, 3,577 participants were recruited via random-digit dialing procedures. Following baseline questionnaires, participants could opt into several follow-up projects. The current investigation merged data from Project 2 (Daily Diary Study, 2012–2014, n = 782) and Project 4 (Biomarker Study, 2012–2016, n = 863) [30]. The analytic sample included 323 individuals who participated in both follow-up projects and had biological aging measures available. As participants in our analytic sample were required to complete both the Daily Diary (Project 2) and Biomarkers (Project 4), they represent a subset selected for high participant engagement. However, prior work on the MIDUS Refresher cohort found that participants completing the Biomarkers assessment (Project 4) were largely comparable to those completing only the broader MIDUS survey components, with similar distributions of age, sex, and race [31]. Detailed descriptions of all MIDUS procedures have been outlined in previous publications [29,30,32,33]. We adhered to the Strengthening the Reporting of Observational Studies in Epidemiology guidelines [34].

### Epigenetic aging

In the MIDUS Refresher Biomarker Study (Project 4), participants completed biomarker assessments across a two-day clinic visit at the University of Wisconsin-Madison, Georgetown University, or the University of California, Los Angeles [30]. The two-day protocol was a standardized feature of the parent study design, enabling fasting blood collection on the morning of Day 2 following an overnight 12-hour urine specimen collection. Whole blood samples were obtained in EDTA-containing BD Vacutainer Tubes before being frozen for storage [35]. Following DNA extraction, samples underwent quality assessment for yield and purity prior to methylation analysis. Genome-wide DNA methylation was measured using the Illumina Methylation EPIC platform. Raw methylation intensity data were processed using the noob background correction method from the minfi R package to minimize technical variation [36]. This processing generated beta values representing methylation percentages at individual CpG sites, which underwent normalization procedures. Data were then mapped to CpG sites compatible with the Illumina 450K array format to ensure compatibility with established epigenetic aging algorithms. Comprehensive quality control measures were applied, including probe detection p-value evaluation, sample call rate assessment, sex concordance checking, and comparison with reference methylation profiles. All samples satisfied quality control standards.

Epigenetic aging biomarkers were calculated from the processed methylation data using two validated algorithms: GrimAge2 and DunedinPACE [17,18]. These specific measures were chosen over other epigenetic clocks available in MIDUS (including Horvath and Hannum) [37,38] because they capture distinct, clinically relevant dimensions of biological aging that were most closely aligned with the aims of the present study [39]. DunedinPACE quantifies the rate of biological aging (years of physiological decline per calendar year), while GrimAge2 estimates cumulative biological damage and predicts mortality and morbidity risk [17,18]. GrimAge2 and DunedinPACE values were standardized to z-scores (mean = 0, standard deviation [SD] = 1) to facilitate comparison of regression coefficients across outcomes, given that these clocks have different measurement scales (years of biological age acceleration and pace of aging per chronological year, respectively).

### Social media use duration

The MIDUS Refresher Daily Diary Project (Project 2) collected daily experiences data through brief telephone interviews administered over eight consecutive days, focusing on self-reported measures of stress and well-being [33]. Daily Diary data collection was conducted throughout the year in small groups of approximately 20 participants, with each group completing the eight-day interview sequence over the same time period [40]. Distributing data collection across the calendar year helped reduce the likelihood that findings would reflect season-specific patterns in daily experiences of social media use. Retention was high: 93% of participants completed at least six days, 90% completed at least seven days, and 80% completed all eight days [41].

During each daily interview, participants were asked “Since this (time/we spoke) yesterday, how much time did you spend on social media websites (Facebook, Twitter, MySpace)?” For data processing, we converted all time reports to minutes and calculated the total minutes of social media use for each day [42]. We then computed each participant’s average daily social media use across all completed study days. Social media use duration was divided by 60 to facilitate interpretation of results as the change in SD units of epigenetic aging per one-hour increase in average daily social media use.

### Covariates

Covariates included inter-assessment interval, sociodemographic characteristics, health behaviors, mental health, and body mass index (BMI). To account for the temporal distance between assessments, we derived the inter-assessment interval as the absolute difference in months between each participant’s daily diary interview date and biomarker collection date. Sociodemographic characteristics included age (continuous variable), sex (male versus female), race/ethnicity (White versus non-White), marital status (married, divorced/separated/widowed, never married), educational attainment (less than college versus college degree or higher) and annual household income from wages (<$50,000 versus $50,000+). Due to small sample sizes, the non-White category comprised participants identifying as Hispanic, Asian, Black, Native American/Alaskan Native, and Native Hawaiian/Pacific Islander.

Health behaviors included smoking status (never, past, current), past month alcohol consumption (never, less than one day per week, one to two days per week, three or more days per week), and leisure-time physical activity. Leisure-time physical activity was operationalized as a composite of moderate and vigorous activity frequency averaged across summer and winter seasons. Each item was rated on a 6-point scale ranging from *1 (several times a week)* to *6 (never)*. Items were reverse-coded prior to averaging such that higher composite scores reflect greater leisure-time physical activity (Cronbach’s α = 0.92). Mental health was assessed using self-reported indicators of depression (yes/no) and anxiety disorder (yes/no) [43]. BMI (kg/m^2^) was obtained from the biomarker assessment and analyzed as a continuous variable.

### Analytic strategy

Zero-order correlations were computed among key study variables (age, social media use duration, GrimAge2, and DunedinPACE). To examine the association between social media use and epigenetic aging, we fit linear regression models in a stepwise manner for each outcome (DunedinPACE and GrimAge2). Model 1 adjusted for inter-assessment interval and sociodemographic characteristics (age, sex, race/ethnicity, marital status, educational attainment, and annual household income). Model 2 added health behaviors (smoking status, alcohol consumption, and leisure-time physical activity). Model 3 further adjusted for mental health (depression and anxiety disorder). Model 4 additionally adjusted for BMI.

Missing data were addressed using multiple imputation by chained equations via the mice R package [44]. We specified logistic regression for race/ethnicity and annual household income, multinomial logistic regression for marital status, and predictive mean matching for leisure-time physical activity. Results were pooled across ten imputed datasets according to Rubin’s rules [45]. All analyses were conducted in R [46], with statistical significance set at *p* < 0.05 (two-sided).

To assess the robustness of primary findings, we conducted three sensitivity analyses. First, we tested for a curvilinear relationship by adding a quadratic term for social media use duration. Second, we modeled a natural log-transformed version of the exposure to account for its skewed distribution. Third, we varied the number of imputed datasets between 20 and 50 to evaluate the stability of results under different multiple imputation specifications.

## Results

### Sample characteristics

The mean age of the sample was 47.5 years (SD = 11.8) and 53.6% of participants were female, 80.2% were White, and 63.2% were married (Table 1). Regarding socioeconomic status, 58.5% had a college degree or higher and 57.6% had an annual household income of at least $50,000. The distribution for self-reported mental health was 13.6% with depression and 3.1% with anxiety disorder. Concerning health behaviors, 69.0% never smoked, 30.0% never consumed alcohol, and the mean leisure-time physical activity score was 4.5 (SD = 1.5). The average BMI was 29.8 kg/m^2^ (SD = 7.4). On average, participants spent 25.5 minutes per day on social media (SD = 54.7). The zero-order correlations are presented in Table 2, with chronological age being significantly correlated with DunedinPACE (r = 0.32, *p* < 0.001) and GrimAge2 (r = 0.89, *p* < 0.001). Social media use duration significantly correlated with DunedinPACE (r = 0.18, *p* = 0.001) but not with GrimAge2 (r = -0.02, *p* = 0.67).

**Table 1:** Characteristics of 323 participants from the midlife in the United States refresher study.

Characteristic	N = 323
Age, Mean (SD)	47.5 (11.8)
Sex, n (%)	
Male	150 (46.4)
Female	173 (53.6)
Race/ethnicity, n (%)	
White	259 (80.2)
non-White	60 (18.6)
Missing	4 (1.2)
Marital status, n (%)	
Married	204 (63.2)
Divorced/Separated/Widowed	64 (19.8)
Never married	54 (16.7)
Missing	1 (0.3)
Educational attainment, n (%)	
Less than college	134 (41.5)
College degree or higher	189 (58.5)
Annual household income, n (%)	
<$50,000	122 (37.8)
$50,000+	186 (57.6)
Missing	15 (4.6)
Depression, n (%)	
No	279 (86.4)
Yes	44 (13.6)
Anxiety disorder, n (%)	
No	313 (96.9)
Yes	10 (3.1)
Smoking status, n (%)	
Never	223 (69.0)
Past	72 (22.3)
Current	28 (8.7)
Alcohol consumption, n (%)	
Never	97 (30.0)
<1 day a week	89 (27.6)
1-2 days a week	66 (20.4)
3 + days a week	71 (22.0)
Leisure-time physical activity, Mean (SD)	4.5 (1.5)
Missing, n (%)	2 (0.6)
Body mass index (kg/m^2^), Mean (SD)	29.8 (7.4)
Inter-assessment interval (months), Mean (SD)	14.5 (7.5)
Social media use duration (average daily minutes), Mean (SD)	25.5 (54.7)
GrimAge2, Mean (SD)	58.2 (10.5)
DunedinPACE, Mean (SD)	0.9 (0.1)

**Table 2:** Zero-order correlations among key study variables.

Variable	1	2	3
1. Age			
2. Social media use duration (average daily minutes)	-0.08		
3. GrimAge2	0.89***	-0.02	
4. DunedinPACE	0.32***	0.18**	0.59***

**p < 0.01.

***p < 0.001.

### Associations between social media use duration and epigenetic aging

**Fig 1:**
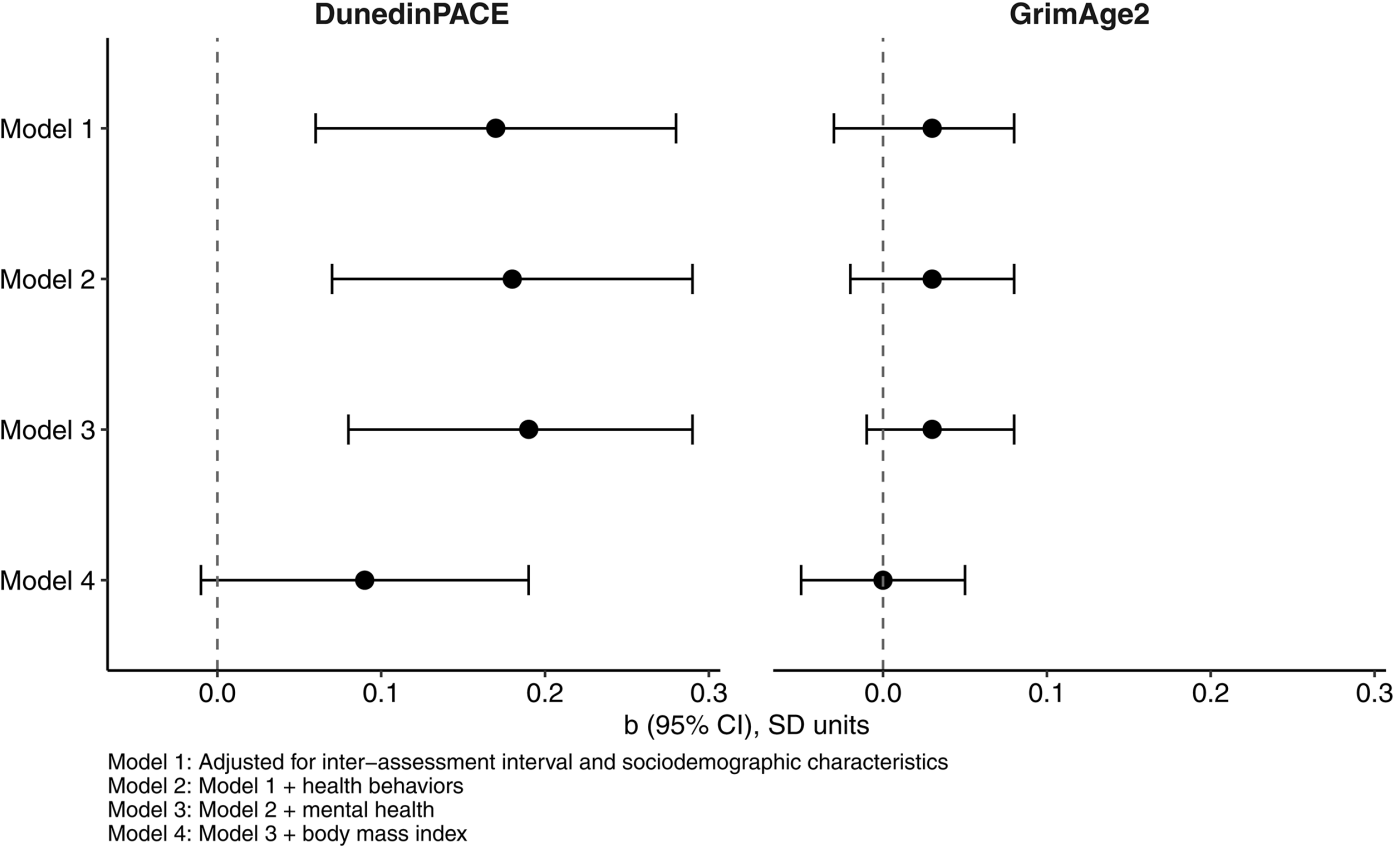
Adjusted associations between social media use duration and epigenetic aging in the Midlife in the United States Refresher Study. Regression coefficients reflect the SD change in epigenetic aging per 1-hour increase in average daily social media use.

Fig 1 and S1 Table show the linear regression models examining the association between social media use duration and epigenetic aging. We generally observed a statistically significant association between social media use duration and DunedinPACE, but not with GrimAge2. Model 1 indicated that each additional hour of social media use was significantly associated with a 0.17 SD increase in DunedinPACE (95% CI: 0.06 to 0.28, *p* = 0.002), adjusting for inter-assessment interval and sociodemographic characteristics. After health behavior adjustment in Model 2, this association slightly increased to a 0.18 SD increase (95% CI: 0.07 to 0.29, *p* = 0.002). The association persisted with additional adjustment for mental health in Model 3 (0.19 SD increase, 95% CI: 0.08 to 0.29, *p* < 0.001). However, introducing BMI in Model 4 attenuated the association to a 0.09 SD increase (95% CI: -0.01 to 0.19, *p* = 0.088). In contrast, we found no evidence of a statistically significant relationship between social media use duration and GrimAge2 across any model specification. Estimates were relatively small and stable across Models 1–3 (0.03 SD increase), with CIs consistently spanning zero. In the final adjusted model additionally controlling for BMI, the association was attenuated to null (0.00 SD change, 95% CI: -0.05 to 0.05, *p* = 0.99). Sensitivity analyses did not support a curvilinear dose-response relationship between social media use duration and epigenetic aging (S2 Table). Findings were also consistent when using a natural log-transformed version of the exposure (S3 Table) and when varying multiple imputation specifications (S4 Table), collectively supporting the robustness of the primary results.

## Discussion

In this national sample of midlife and older U.S. adults, greater social media use duration was associated with a faster pace of biological aging as measured by DunedinPACE. This association persisted after comprehensive adjustment for demographic, socioeconomic, and health-related factors, as well as smoking status and alcohol use. However, when BMI was included, the association was attenuated and no longer statistically significant. In contrast, no association between social media use and GrimAge2 was observed in any model specification.

Our findings extend the emerging conceptualizations of digital environments as contemporary social contexts relevant to health [47,48]. Although social media is frequently examined through psychosocial frameworks [49], the observed attenuation after BMI adjustment suggests that behavioral or metabolic factors related to BMI may also be relevant to the association between social media use duration and DunedinPACE. However, BMI may reflect the downstream consequences of broader lifestyle, psychosocial, environmental, and health-related processes [50] that were not directly measured in the present study. The role of BMI in this association is noteworthy given prior evidence linking BMI with epigenetic age acceleration [51,52], as well as established links between chronic low-grade inflammation, and metabolic dysregulation [53,54]. These findings raise the possibility that metabolic-inflammatory pathways may be relevant to the observed association between social media use and accelerated biological aging as captured by DunedinPACE [53,55–60], although the present study cannot determine whether BMI operates as a mediator, confounder, or marker of shared underlying processes. The absence of an association with GrimAge2 further implies that social media use may relate more closely to the pace of ongoing physiological change than to cumulative mortality-linked methylation signatures that evolve over time [17,18]. This interpretation is consistent with prior evidence suggesting that metabolic and lifestyle-related factors may show different patterns of association across epigenetic measures, with some associations persisting for DunedinPACE after lifestyle adjustment but attenuating for GrimAge [61].

It is important to note, however, that digital engagement is not uniformly detrimental. Online platforms can foster connection, support, and access to health-promoting information [62,63], suggesting that the biological effects of social media use are heterogeneous and likely context-dependent. Thus, the health relevance of social media use likely depends not only on duration, but also on the quality and context of engagement, including whether interactions are primarily supportive, stressful, exclusionary, or socially threatening [64,65]. Moreover, individuals already exposed to chronic stress or social disadvantage offline may experience amplified psychosocial strain in digital environments where social hierarchies and inequities are often reproduced and intensified [66,67].

Overall, this work provides preliminary evidence connecting social media use to accelerated epigenetic aging in a national sample, employing rigorous methods including daily diary assessments and two next-generation DNA methylation clocks. Several methodological strengths merit consideration. The daily diary design reduced reliance on a single retrospective estimate of social media use by capturing duration repeatedly across consecutive days. In addition, the use of complementary epigenetic aging measures enabled assessment of whether associations differed across distinct dimensions of biological aging, including the pace of physiological decline and mortality-linked methylation signatures. While these features are notable strengths, the interpretive scope of our findings is shaped by important limitations. It is also important to note that our sample was predominantly White, limiting the ability to assess racial/ethnic variation in the observed association. Future research with larger and more diverse samples is needed to examine whether associations between social media use and biological aging vary across racial/ethnic groups, age, sex, and BMI. Moreover, the cross-sectional design precludes causal inference and limits interpretation of BMI as either a mediator, confounder, or shared correlate. Accordingly, the present findings should be interpreted as associative rather than directional, and longitudinal studies will be necessary to clarify temporality and the role of behavioral and psychosocial pathways. Furthermore, self-reported usage duration cannot capture qualitative aspects of engagement, such as content, context, or affective tone, that likely moderate biological outcomes. Self-reported measures of social media use are also subject to potential recall error and misclassification, particularly given variability in how individuals define and interpret social media platforms and activities, as well as broader inconsistencies in how social media use is conceptualized and measured across studies [68,69]. Future longitudinal studies integrating objective digital trace data, ecological momentary assessment, and mechanistic biomarkers (e.g., cortisol, cytokines) will be essential to delineate causal pathways linking digital contexts with aging processes.

## Conclusion

These findings provide preliminary evidence that social media use may be linked to biological aging processes, particularly those captured by methylation measures of the pace of biological aging such as DunedinPACE. The attenuation of effects after accounting for BMI suggests that behavioral, metabolic, and lifestyle-related processes may be relevant to this association. By integrating digital behavior into models of health and aging, this work highlights the potential importance of considering online environments as meaningful components of contemporary social and behavioral contexts. As digital platforms increasingly structure daily interactions, clarifying how they influence behavioral, psychosocial, and physiological processes will be essential to advancing biobehavioral theories of stress, adaptation, and aging.

## Supporting information

S1 Table Adjusted associations between social media use duration and epigenetic aging in the midlife in the United States refresher study. (DOCX)

S2 Table Linear and quadratic associations between social media use duration and epigenetic aging in the midlife in the United States refresher study. (DOCX)

S3 Table Adjusted associations between natural log-transformed social media use duration and epigenetic aging in the midlife in the United States refresher study. (DOCX)

S4 Table Adjusted associations between social media use duration and epigenetic aging in the midlife in the United States refresher study (pooled across 20 and 50 imputed datasets). (DOCX)
