# Supplementary material for: Social media use duration and epigenetic aging among U.S. adults in the MIDUS refresher study
Source: PLOS Digit Health. 2026 Aug 6;5(8):e0001570. doi: 10.1371/journal.pdig.0001570 (PMC13446696; doi:10.1371/journal.pdig.0001570)
Supplement: Supplementary file 1 [file pdig.0001570.s001.docx]

**S1 Table.** Adjusted associations between social media use duration and epigenetic aging in the Midlife in the United States Refresher Study.

|  | **DunedinPACE** | | **GrimAge2** | |
| --- | --- | --- | --- | --- |
| **Characteristic** | **Beta** **(95% CI)** | **p-value** | **Beta** **(95% CI)** | **p-value** |
| Model 1 | **0.17 (0.06 to 0.28)** | **0.002** | 0.03 (-0.03 to 0.08) | 0.30 |
| Model 2 | **0.18 (0.07 to 0.29)** | **0.002** | 0.03 (-0.02 to 0.08) | 0.26 |
| Model 3 | **0.19 (0.08 to 0.29)** | **<0.001** | 0.03 (-0.01 to 0.08) | 0.16 |
| Model 4 | 0.09 (-0.01 to 0.19) | 0.088 | 0.00 (-0.05 to 0.05) | 0.99 |
| Abbreviation: CI = Confidence Interval | | | | |
| Results were pooled across ten imputed datasets. | | | | |
| Bold indicates *p*<0.05. | | | | |
| Model 1: Adjusted for inter-assessment interval and sociodemographic characteristics | | | | |
| Model 2: Model 1 + health behaviors | | | | |
| Model 3: Model 2 + mental health | | | | |
| Model 4: Model 3 + body mass index | | | | |
