# Supplementary material for: Social media use duration and epigenetic aging among U.S. adults in the MIDUS refresher study
Source: PLOS Digit Health. 2026 Aug 6;5(8):e0001570. doi: 10.1371/journal.pdig.0001570 (PMC13446696; doi:10.1371/journal.pdig.0001570)
Supplement: Supplementary file 2 [file pdig.0001570.s002.docx]

**S2 Table.** Linear and quadratic associations between social media use duration and epigenetic aging in the Midlife in the United States Refresher Study.

|  | **DunedinPACE** | | **GrimAge2** | |  |
| --- | --- | --- | --- | --- | --- |
| **Characteristic** | **Beta** **(95% CI)** | **p-value** | **Beta** **(95% CI)** | **p-value** |  |
| Model 1 (linear) | 0.20 (-0.02 to 0.42) | 0.070 | 0.03 (-0.07 to 0.14) | 0.54 |  |
| Model 1 (quadratic) | 0.00 (-0.03 to 0.02) | 0.77 | 0.00 (-0.01 to 0.01) | 0.91 |  |
| Model 2 (linear) | 0.20 (-0.01 to 0.42) | 0.064 | 0.03 (-0.07 to 0.14) | 0.52 |  |
| Model 2 (quadratic) | 0.00 (-0.03 to 0.02) | 0.80 | 0.00 (-0.01 to 0.01) | 0.93 |  |
| Model 3 (linear) | **0.26 (0.05 to 0.47)** | **0.015** | 0.04 (-0.06 to 0.13) | 0.42 |  |
| Model 3 (quadratic) | -0.01 (-0.03 to 0.02) | 0.44 | 0.00 (-0.01 to 0.01) | 0.93 |  |
| Model 4 (linear) | 0.15 (-0.04 to 0.35) | 0.13 | 0.00 (-0.09 to 0.09) | 0.99 |  |
| Model 4 (quadratic) | -0.01 (-0.03 to 0.01) | 0.46 | 0.00 (-0.01 to 0.01) | 0.98 |  |
| Abbreviation: CI = Confidence Interval | | | | | |
| Results were pooled across ten imputed datasets. | | | | | |
| Bold indicates *p*<0.05. | | | | | |
| Model 1: Adjusted for inter-assessment interval and sociodemographic characteristics | | | | | |
| Model 2: Model 1 + health behaviors | | | | | |
| Model 3: Model 2 + mental health | | | | | |
| Model 4: Model 3 + body mass index | | | | | |
