# Supplementary material for: Social media use duration and epigenetic aging among U.S. adults in the MIDUS refresher study
Source: PLOS Digit Health. 2026 Aug 6;5(8):e0001570. doi: 10.1371/journal.pdig.0001570 (PMC13446696; doi:10.1371/journal.pdig.0001570)
Supplement: Supplementary file 3 [file pdig.0001570.s003.docx]

**S3 Table.** Adjusted associations between natural log-transformed social media use duration and epigenetic aging in the Midlife in the United States Refresher Study.

|  | **DunedinPACE** | | **GrimAge2** | |
| --- | --- | --- | --- | --- |
| **Characteristic** | **Beta** **(95% CI)** | **p-value** | **Beta** **(95% CI)** | **p-value** |
| Model 1 | **0.40 (0.12 to 0.69)** | **0.006** | 0.05 (-0.09 to 0.18) | 0.50 |
| Model 2 | **0.42 (0.13 to 0.71)** | **0.004** | 0.05 (-0.08 to 0.19) | 0.45 |
| Model 3 | **0.47 (0.19 to 0.74)** | **<0.001** | 0.06 (-0.06 to 0.18) | 0.32 |
| Model 4 | 0.22 (-0.04 to 0.48) | 0.10 | -0.02 (-0.15 to 0.10) | 0.69 |
| Abbreviation: CI = Confidence Interval | | | | |
| Results were pooled across ten imputed datasets. | | | | |
| Bold indicates *p*<0.05. | | | | |
| Model 1: Adjusted for inter-assessment interval and sociodemographic characteristics | | | | |
| Model 2: Model 1 + health behaviors | | | | |
| Model 3: Model 2 + mental health | | | | |
| Model 4: Model 3 + body mass index | | | | |
