# Supplementary material for: Social media use duration and epigenetic aging among U.S. adults in the MIDUS refresher study
Source: PLOS Digit Health. 2026 Aug 6;5(8):e0001570. doi: 10.1371/journal.pdig.0001570 (PMC13446696; doi:10.1371/journal.pdig.0001570)
Supplement: Supplementary file 4 [file pdig.0001570.s004.docx]

**S4 Table.** Adjusted associations between social media use duration and epigenetic aging in the Midlife in the United States Refresher Study (pooled across 20 and 50 imputed datasets).

|  | **DunedinPACE** | | **GrimAge2** | |
| --- | --- | --- | --- | --- |
| *Results pooled across 20 imputed datasets.* | | | | |
| **Characteristic** | **Beta** **(95% CI)** | **p-value** | **Beta** **(95% CI)** | **p-value** |
| Model 1 | **0.17 (0.06 to 0.28)** | **0.003** | 0.03 (-0.03 to 0.08) | 0.31 |
| Model 2 | **0.18 (0.07 to 0.29)** | **0.002** | 0.03 (-0.02 to 0.08) | 0.27 |
| Model 3 | **0.19 (0.08 to 0.29)** | **<0.001** | 0.03 (-0.01 to 0.08) | 0.16 |
| Model 4 | 0.09 (-0.01 to 0.19) | 0.089 | 0.00 (-0.05 to 0.05) | 0.98 |
| *Results pooled across 50 imputed datasets.* | | | | |
| **Characteristic** | **Beta** **(95% CI)** | **p-value** | **Beta** **(95% CI)** | **p-value** |
| Model 1 | **0.17 (0.06 to 0.28)** | **0.003** | 0.03 (-0.03 to 0.08) | 0.32 |
| Model 2 | **0.18 (0.07 to 0.29)** | **0.002** | 0.03 (-0.02 to 0.08) | 0.27 |
| Model 3 | **0.19 (0.08 to 0.29)** | **<0.001** | 0.03 (-0.01 to 0.08) | 0.17 |
| Model 4 | 0.09 (-0.01 to 0.19) | 0.090 | 0.00 (-0.05 to 0.05) | 0.97 |
| Abbreviation: CI = Confidence Interval | | | | |
| Bold indicates *p*<0.05. | | | | |
| Model 1: Adjusted for inter-assessment interval and sociodemographic characteristics | | | | |
| Model 2: Model 1 + health behaviors | | | | |
| Model 3: Model 2 + mental health | | | | |
| Model 4: Model 3 + body mass index | | | | |
